# Metagenomic Next-Generation Sequencing Improves Diagnosis of Osteoarticular Infections From Abscess Specimens: A Multicenter Retrospective Study

**DOI:** 10.3389/fmicb.2020.02034

**Published:** 2020-09-15

**Authors:** Mingwei Zhao, Kai Tang, Fengsheng Liu, Weidong Zhou, Jun Fan, Guangxuan Yan, Shibing Qin, Yu Pang

**Affiliations:** ^1^Department of Orthopedics, Qingdao Chest Hospital, Qingdao, China; ^2^Department of Orthopedics, Beijing Chest Hospital, Capital Medical University, Beijing Tuberculosis and Thoracic Tumor Research Institute, Beijing, China; ^3^Department of Orthopedics, The Chest Hospital of Hebei Province, Shijiazhuang, China; ^4^National Clinical Laboratory on Tuberculosis, Beijing Chest Hospital, Capital Medical University, Beijing Tuberculosis and Thoracic Tumor Research Institute, Beijing, China

**Keywords:** metagenomic, osteoarticular, infection, diagnosis, metagenomic next-generation sequencing

## Abstract

**Background**: We conducted this retrospective study to reveal the accuracy of metagenomic next-generation sequencing (mNGS) for diagnosing osteoarticular infections from fresh abscess specimens obtained from patients in an HIV-naive population.

**Methods**: We retrospectively analyzed hospital records at three participating TB-specialized hospitals for patients admitted with suggestive diagnoses of osteoarticular tuberculosis between January 2018 and August 2019. Abscess specimens obtained from each patient were tested *via* pathogen culture, GeneXpert *Mycobacterium tuberculosis* (MTB)/rifampicin (RIF), and mNGS assay.

**Results**: A total of 82 abscess samples were collected from patients with osteoarticular infections, including 53 cases with (64.6%) bacterial, 21 (25.6%) with mycobacterial, 7 (8.5%) with fungal, and 1 (1.2%) with actinomycetal organisms detected. Analysis of mNGS assay results identified potential pathogens in all cases, with *M. tuberculosis* complex (MTBC) most frequently isolated, followed by *Staphylococcus aureus* and *Brucella melitensis*. Conventional culture testing identified causative pathogens in only 48.4% of samples, a significantly lower rate than the mNGS pathogen identification rate (100%, *p* < 0.01). Culture-positive group specimens yielded significantly greater numbers of sequence reads than did culture-negative group specimens (*p* < 0.01). Of patients receiving surgical interventions and mNGS-guided treatment, 76 (92.7%) experienced favorable outcomes by the time of follow-up assessment at 3 months post-treatment. Notably, MTBC detection in two patients experiencing treatment failure suggests that they had mixed infections with MTBC and other pathogens.

**Conclusion**: Results presented here demonstrate that mNGS has a greater pathogen detection rate in osteoarticular infections than conventional culture-based methods.

## Introduction

Bone and joint infections are generally common worldwide and cause considerable patient morbidity (Quick et al., 2018). Despite ongoing medical advances, a high proportion of patients report persistent disability, limited function, and lower quality of life, especially those infected with tubercle bacilli (Wilson and Winn, 2008). Osteoarticular tuberculosis (TB), which currently accounts for ~15% of extrapulmonary TB cases in developed countries and ~40% in China, usually leads to death or long-term disability (Pigrau-Serrallach and Rodriguez-Pardo, 2013). Patients with comorbidities, especially immunocompromised patients, experience worse outcomes than otherwise healthy patients (Quick et al., 2018). Importantly, surgical interventions are often required for treatment of osteoarticular infections, with medication-based therapies administered prior to surgery to completely eliminate bacteria within lesions. Therefore, early and accurate pathogen diagnosis is essential before formulating effective chemotherapeutic regimens, while avoiding empirical treatment-associated toxicity (Besser et al., 2018).

Abscess tissue is the predominant specimen type tested for identification of causative pathogens of osteoarticular infections (Ma et al., 2019). As the default host response to bacterial invasion, abscess formation entails neutrophilic destruction of pathogens, with the resulting abscess comprised mainly of accumulated dead neutrophils remaining in tissues after clearance of invading bacteria (Kahn and Pritzker, 1973; Miller and Cho, 2011). Bacterial culture-based testing, considered the gold standard for detection of bacteria in abscesses, requires live replicating cells and requires days to months of time before yielding results, depending on the growth rate of bacteria within infection sites (Brook, 2008). However, intra-abscess interactions between immunocytes and pathogenic bacteria significantly reduce bacterial viability, leading to unacceptably lower bacterial recovery than that needed to obtain reliable culture-based test results (Miller and Cho, 2011). Meanwhile, numerous PCR-based molecular diagnostic tests offer moderate sensitivity for bacterial detection (Tarkin et al., 2005). Importantly, the use of such PCR-based tests has demonstrated that a negative result obtained using culture-based methods cannot serve as reliable evidence, demonstrating the absence of bacteria within a sample. However, PCR-based tests specifically targeting a species or group of species cannot be used effectively without some prior knowledge regarding the identity of the pathogen in question. Thus, there is an urgent need to develop new bacterial species-specific diagnostic tests for rapid and accurate identification of infection-site pathogens.

Novel metagenomic next-generation sequencing (mNGS) platforms now permit rapid pathogen detection and species identification from a single clinical sample (Simner et al., 2018). Notably, this non-targeted identification strategy constitutes a revolutionized shift in microbiological diagnosis away from conventional molecular diagnostic test strategies based on prior assumptions regarding infecting organisms (Besser et al., 2018). Another important benefit of mNGS is its shortened turnaround time of 2 days, enabling timely initiation of effective treatment (Besser et al., 2018). Consequently, infectious disease clinicians have used mNGS frequently for detection of pathogens in various clinical specimens, such as blood, cerebrospinal fluid, and bronchoalveolar lavage fluid (Parize et al., 2017; Miller et al., 2019; Li et al., 2020). Indeed, mNGS often provides an alternative choice to diagnose patients with suspected infections for which the pathogenic cause is unknown in spite of prior comprehensive microbiological investigations. Nevertheless, to our knowledge, only limited data have been reported regarding clinical use of mNGS for identification of pathogens within osteoarticular infection-associated abscesses despite numerous reports (Ruppe et al., 2017). As a consequence, this study aimed to evaluate the retrospective diagnostic accuracy of mNGS for diagnosing osteoarticular infections using fresh abscess specimens from an HIV-naive patient population.

## Materials and Methods

### Setting

Inpatients were selected from three participating TB-specialized hospitals: Beijing Chest Hospital, Qingdao Chest Hospital, and Hebei Chest Hospital. We retrospectively analyzed hospital records and then selected patients admitted between January 2018 and August 2019 with symptoms suggestive of osteoarticular tuberculosis that included: (i) fever, (ii) the presence of osteoarticular pain not relieved by rest or analgesics, (iii) magnetic resonance imaging (MRI) abnormalities, and (iv) previous and current TB episodes. One abscess specimen was surgically biopsied from each patient and submitted to clinical laboratories for pathogen culture and GeneXpert *Mycobacterium tuberculosis* (MTB)/rifampicin (RIF) assay testing. All three institutions followed the same diagnostic algorithm: the mNGS assay was used to test all patients after they consented to mNGS testing in spite of its high out-of-pocket cost. After patients provided informed written consent to undergo surgery and mNGS assay testing, abscess tissue specimens were collected and then processed immediately for mNGS. After acquisition of mNGS data from abscess biopsies, physicians supervising patient treatment employed mNGS assay-based pathogen identification to guide treatment of osteoarticular infections (referred to as mNGS-guided therapy). After completion of mNGS-guided therapy, patients were evaluated for clinical outcomes at 3 months post-treatment completion. Based on outcome definitions used in a previous study, a favorable outcome was defined as follows: (1) resolution of clinical symptoms due to infection, (2) improvement of osteoarticular function, and (3) improvement of inflammation, as indicated by inflammatory biomarkers and radiological features (Wieland et al., 2012). This study was approved by the Ethics Committee of Qingdao Chest Hospital (QXLL2019003). Each participant signed a written informed consent form agreeing to use of anonymized clinical data.

### Laboratory Examination

After abscess specimen collection, specimens were tested *via* smear microscopy, mycobacterial culture, aerobic bacterial culture, anaerobic bacterial culture, and GeneXpert MTB/RIF assay. Briefly, smear microscopy was conducted following guidelines of the National Tuberculosis Control Plan (Xia et al., 2013). One milliliter of each abscess sample was treated for 15 min with N-acetyl-L-cysteine and sodium hydroxide (NALC-NaOH). After neutralization with phosphate buffer (PBS) and centrifugation for 15 min at 3,000 × *g*, each pellet was inoculated into a BACTEC mycobacteria growth indicator tube (MGIT; BD Microbiology Systems, United States; Ma et al., 2019). In addition, abscess specimens were plated and incubated for up to 5 days on 5% sheep blood and MacConkey agar for aerobic culture and 5% sheep blood agar for anaerobic culture. Finally, GeneXpert MTB/RIF was conducted within 2 h to detect the presence of *M. tuberculosis* DNA in abscess samples, in accordance with the manufacturer’s instructions. The remaining material in each specimen was analyzed using mNGS assay.

### Metagenomic Next-Generation Sequencing

Abscess samples (a 600-μl volume of each) from all patients were each mixed with 1 g of 0.5-mm diameter glass beads and then placed on a vortex mixer for 30 min at 3,000 rpm. Next, 300 μl of each pretreated sample was subjected to DNA extraction using the TIANamp Micro DNA Kit (Tiangen Biotech, Beijing, China) according to the manufacturer’s instructions. Purified DNA was fragmented into 200–300 bp segments using ultrasound followed by end-repair, ligation with multiplex barcode adapters, and PCR amplification to complete construction of DNA libraries. After molarities of DNA libraries were estimated using indexing PCR, DNA concentrations were determined *via* the DNA Qubit Assay (Thermo Fisher); meanwhile, DNA quality was evaluated electrophoretically using an Agilent 2,100 system (Agilent Technologies, Santa Clara, CA). Up to 20 qualified DNA libraries were pooled, and then pooled libraries were subjected to DNA sequencing analysis using the MGISEQ-2000 platform.

### Bioinformatics Analysis

Low-quality sequences and adaptor sequences were first removed to generate clean reads. Subsequently, sequences mapping to the human reference genome (hg19) were subtracted from clean reads using Burrows-Wheeler Alignment software (version 0.7.10-r789). Nonhuman sequence reads from each sample were submitted to the Genome Sequence Archive of the Beijing Institute of Genomics, Chinese Academy of Sciences^1^ under accession number PRJCA000880. Additionally, the remaining data were further mapped against the RefSeq Microbial Genome Database of viruses, bacteria, fungi, and parasites created and maintained by the National Center of Biotechnology Information^2^. RefSeq analysis yielded 1,798 whole genome sequences matching those of DNA virus taxa, 6,350 bacterial genomes or scaffolds, 1,064 pathogenic fungi of human infections, and 234 parasites associated with human diseases (Wang et al., 2019). Reporting criteria for infectious pathogens identified by mNGS included: (i) >30% relative abundance at the genus level in bacteria or fungi; (ii) at least three unique reads from a single viral, bacterial, or fungal species; (iii) at least one unique read matching *M. tuberculosis* complex (MTBC) species (Wang et al., 2019). If more than one pathogen was detected, the species present in greatest relative abundance yielding the highest number of unique reads was deemed the probable species associated with osteoarticular infection in that patient.

### Statistical Analysis

Study subject demographic and clinical data were collected by reviewing electronic medical records. Data included gender, age, comorbidities, clinical symptoms, laboratory results, radiological features, and treatment regimens. All data were entered using double manual data entry using the EpiData Entry program, version 3.1 (EpiData Association, Odense, Denmark). Statistical analysis was performed using SPSS software, version 20.0 (IBM SPSS, Chicago, Illinois). Chi-square test and Fisher exact test were used for categorical variables, while *t*-test or Mann-Whitney U test was used for continuous variables, as appropriate. A two-sided value of *p* < 0.05 was considered statistically significant.

## Results

### Participants

A total of 82 abscess samples were collected from patients with osteoarticular infections. Subject demographics, clinical symptoms, and laboratory testing information are summarized in Table 1. Of these 82 patients, 52 were male. The mean age was 54.6 years (range 12.0–90.0 years). All patients experienced pain at infected sites, with only 48.8% of patients self-reporting fever prior to hospital admission. The most frequent comorbidities observed were hypoalbuminemia (17.1%) and diabetes (15.9%).

**Table 1:** Characteristics of individuals enrolled in the study.

Characteristic^a^	No. of patients (%) (*N* = 82)
Age, y, mean (range)	54.6 (12.0–90.0)
Sex, male	52 (63.4)
**Site of infection**			
Spine	60 (73.2)
Joint and others	22 (26.8)
**Symptom**			
Pain	82 (100.0)
Fever	40 (48.8)
Weight loss	2 (2.4)
Night sweat	4 (4.9)
**Laboratory parameters, median (range)^b^**			
WBC count, 10^9^ cells/L	8.3 (3.0–23.0)
ESR, mm/h	71.1 (2.0–149.0)
CRP, mg/L	58.6 (1.4–220.0)
**Comorbidity**			
Diabetes	13 (15.9)
Immunosuppression	8 (9.8)
Hepatic dysfunction	3 (3.7)
Hypoalbuminemia	14 (17.1)
Others	8 (9.8)

a WBC, white blood cell; ESR, erythrocyte sedimentation rate; CRP, C-reactive protein.

b The reference ranges for laboratory parameters: WBC, 4.0–10.0 × 10^9^ cells/L; ESR, 0–20 mm/h; CRP, 0–8.0 mg/L.

### mNGS Assay

**Figure 1:**
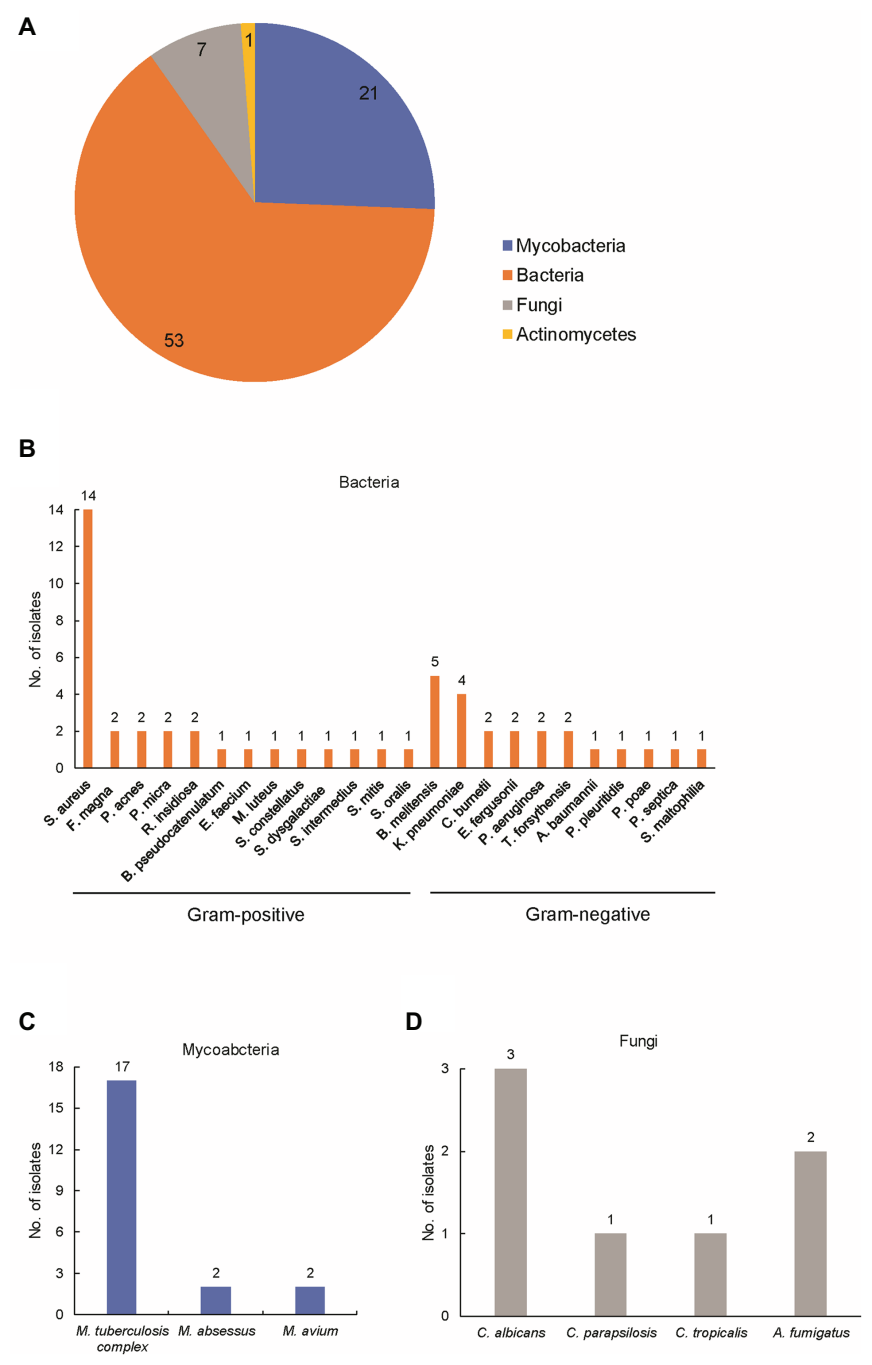
Detection of pathogens by metagenomic next-generation sequencing (mNGS) in patients enrolled in this study. **(A)** Composition of pathogens stratified to bacteria, mycobacteria, fungi, and actinomycetes. The number represents the cases infected with the corresponding pathogen; **(B)** composition of bacteria in patients enrolled in this study; **(C)** composition of mycobacteria in patients enrolled in this study; and **(D)** composition of actinomycetes in patients enrolled in this study.

Using optimized procedures, we conducted mNGS on 82 abscess samples obtained from the 82 patients. Numbers of raw sequence reads ranged from 3.1 × 10^6^ to 5.0 × 10^7^ reads, with a median of 2.4 × 10^7^ reads per abscess specimen. As shown in Figure 1, the mNGS assay could identify potential osteoarticular infection-associated pathogens in all cases, including 53 cases with (64.6%) bacterial, 21 (25.6%) with mycobacterial, 7 (8.5%) with fungal, and 1 (1.2%) with actinomycetal organisms detected. Sequence read numbers obtained for each pathogen ranged from 1 to 5.7 × 10^5^ reads per sample (Supplementary Table S1). These 82 pathogens were classified into 32 species. The most frequent species detected was MTBC (17/82, 20.7%), followed by *Staphylococcus aureus* (14/82, 17.0%) and *Brucella melitensis* (5/82, 6.1%; Supplementary Table S1). Using conventional bacterial culture plus GeneXpert as combined reference standard, the sensitivity of mNGS assay was 100.0% [45/45, 95% confidence interval (CI): 100.0–100.0%].

### Comparison of mNGS Versus Conventional Culture

**Figure 2:**
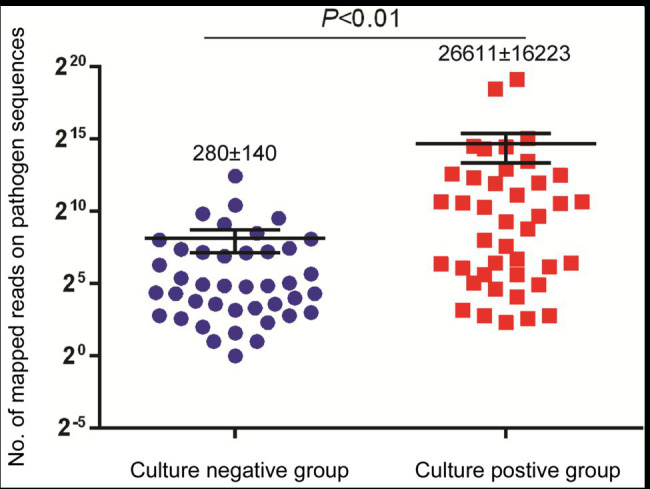
Comparison of sequencing reads between culture-negative and culture positive cases.

We further compared the performance of mNGS vs. conventional culture testing after stratifying results by pathogen type (Table 2). Only 43.4% (23/53) and 57.1% (12/21) of bacterial and mycobacterial organisms were detected in abscess specimens, respectively, based on positive growth results of culture-based testing as compared to mNGS assay results (*p* < 0.01). When comparing results of conventional culture testing to overall pathogen detection results obtained here from all laboratory tests across all samples, conventional culture testing identified causative pathogens in only 40 samples, for a detection rate of 48.4% (95% CI: 48.0–59.6); this rate was significantly lower than that of the mNGS assay (100.0%, 95% CI: 100.0–100.0; *p* < 0.01). Meanwhile, we also found that specimens of the culture-positive group (280 ± 140 reads) yielded significantly higher numbers of sequence reads than did culture-negative group specimens (26,611 ± 16,223 reads, *p* < 0.01; Figure 2).

**Table 2:** Performance of mNGS vs. laboratory culture.

Classification of infections	No. of samples	Organisms identified by mNGS (%)^a^	Organisms identified by culture (%)	*p* value
Bacteria	53	53 (100.0)	23 (43.4)	<0.01
Mycobacteria	21	21 (100.0)	12 (57.1)	0.01
Fungi	7	7 (100.0)	5 (71.4)	0.46
Actinomycetes	1	1 (100.0)	0 (0.0)	-
Total	82	82 (100.0)	40 (48.8)	<0.01

a mNGS, metagenomic next generation sequencing.

**Figure 3:**
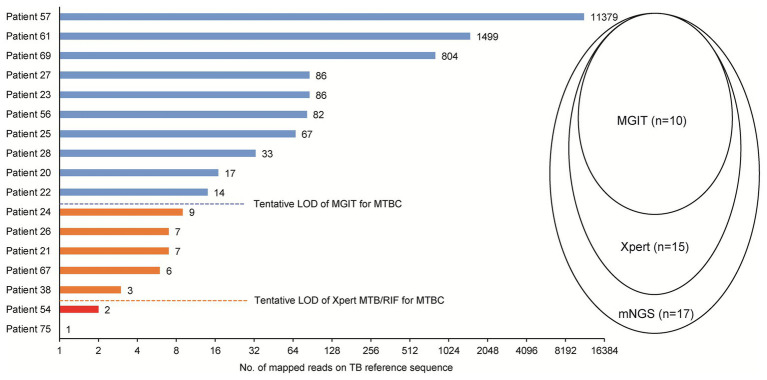
Comparison of mNGS, mycobacterial culture, and Xpert *Mycobacterium tuberculosis* (MTB)/rifampicin (RIF) for detection of MTB in abscess specimens. LOD, limit of detection.

Of 17 tuberculosis cases, 10 were scored as culture-positive cases when assessed by MGIT, for a detection rate of 58.8%. Using the Xpert assay, an additional five cases were detected, for a detection rate of 88.2%. Correlations between sequence reads and results obtained using different methods among TB cases detected here are shown in Figure 3. Notably, the minimum number of sequence reads needed for successful detection using MGIT was >10 sequence reads, while for the Xpert assay the corresponding lower limit was >3 sequence reads.

### Clinical Outcomes

All patients underwent surgical interventions, yielding osteoarticular abscess biopsy specimens that were tested to identify causative infectious organisms. Based on pathogen identification results, patients received appropriate mNGS-guided medication therapies. Of 82 treated patients, 76 (92.7%) experienced favorable outcomes noted at follow-up at 3 months post-treatment. By contrast, adverse outcomes were noted for 6 (7.3%) patients, including 3 (3.7%) treatment failures and 3 (3.7%) deaths. Of the three patients who died, one died from disseminated meningitis, one from acute renal failure, and one from heart failure (Table 3).

**Table 3:** Treatment outcomes of patients with osteoarticular infections.

Treatment outcome	No. of patients (%)
Favorable outcome	76 (92.7)
Adverse outcome	6 (7.3)
Failure^a^	3 (3.7)
Death^b^	3 (3.7)
Total	82 (100.0)

a Treatment failure represents the patients experiencing recurrent abscesses after the combination of surgical interventions and mNGS-guided medication treatment.

b Death includes one patient died from the disseminated infection, one patient from acute renal failure, and one patient from heart failure.

**Figure 4:**
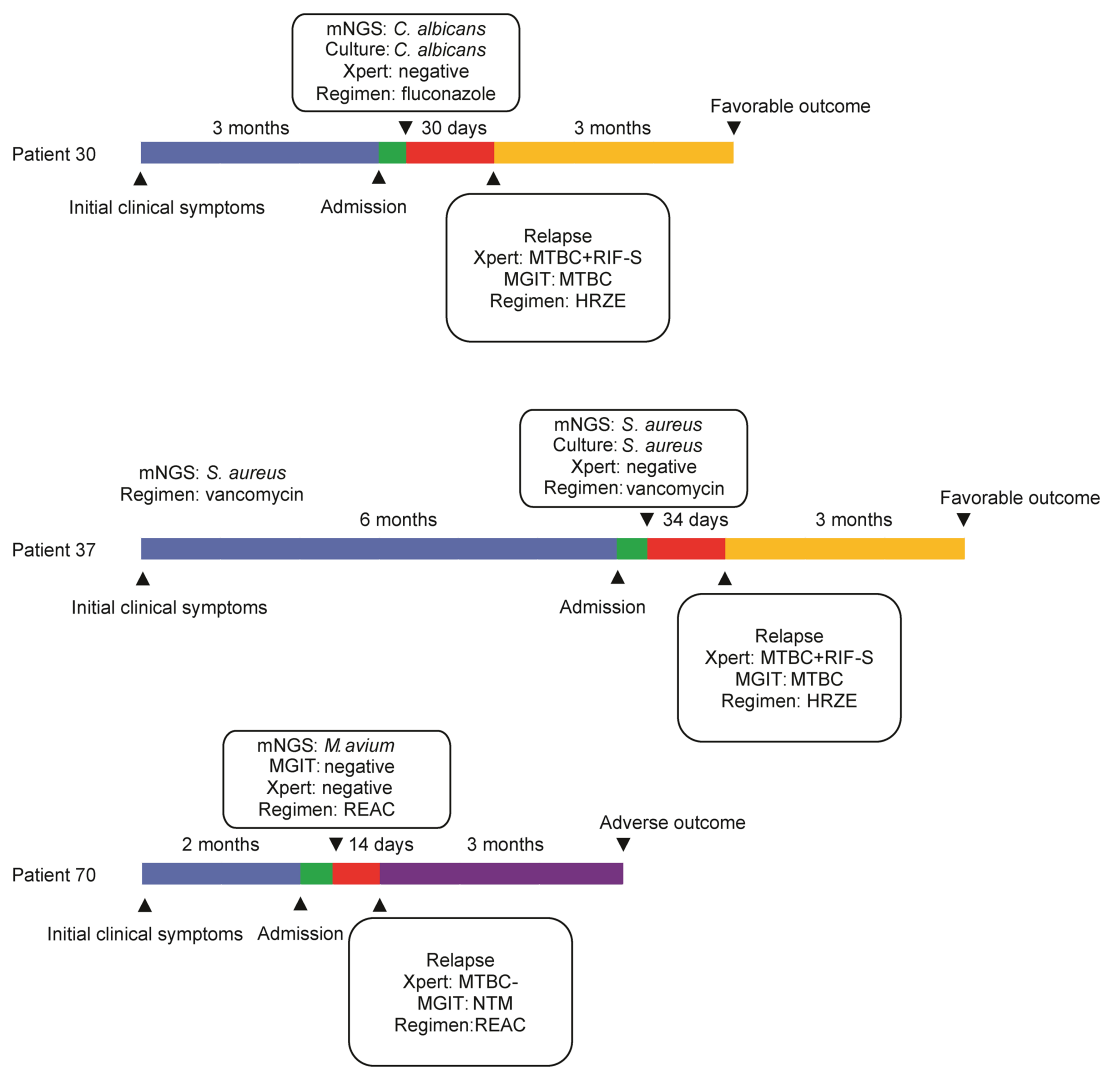
Description of patients suffering from adverse outcomes under the mNGS-guided treatment.

Major clinical events, laboratory findings from abscess tissue testing (including mNGS assay results), and treatment failures throughout the entire treatment course are summarized in Figure 4. Patient 30 experienced aggravated pain for 3 months that was diagnosed using mNGS assay as a *Candida albicans* infection. However, after completion of a 30-day course of fluconazole, as is appropriate for treating presumptive *C. albicans* infection, the abscess re-emerged, and thus was scored as a treatment failure. Meanwhile, mNGS analysis confirming *S. aureus* as causative pathogen for Patient 37 led to treatment with a 34-day course of vancomycin that did not eliminate the infection, and thus led to treatment failure. For Patient 70, the results of mNGS testing revealed *Mycobacterium avium*; however, after administration of RIF, ethambutol, amikacin, and clarithromycin for 14 days, no significant resolution of clinical symptoms was self-reported. For another three patients with adverse outcomes, reanalysis of results of conventional culture and Xpert revealed RIF-susceptible MTB detected by Xpert and MGIT for both Patient 30 and Patient 37, prompting administration of modified anti-infection regimens containing a combination of RIF, isoniazid, ethambutol, and pyrazinamide. Consequently, these patients experienced favorable outcomes at follow-up at 3 months after treatment completion.

## Discussion

Metagenomic next-generation sequencing can accelerate diagnosis of difficult-to-detect pathogens in clinical practice (Thoendel et al., 2018). After applying this approach to abscess specimens, our data demonstrated that mNGS provided significantly greater pathogen detection rate than obtained using conventional culture-based testing. Meanwhile, several recently reported studies have described excellent mNGS assay-based pathogen detection in diverse clinical sample types, such as cerebrospinal fluid (CSF) and bronchoalveolar lavage specimens (Miller et al., 2019; Li et al., 2020). Notably, abscess tissues and CSF are both considered microbiologically sterile (except for infecting pathogens). Thus, mNGS and CSF data should be more easily interpreted than data from nonsterile sites that are normally inhabited by diverse microorganisms. In the latter case, it would be difficult to discriminate between normal colonizing organisms and pathogens when determining the cause of infection. Meanwhile, a previous report comparing the accuracy of mNGS to that of conventional clinical laboratory testing of CSF specimens found that 13 of 84 positive specimens were missed by mNGS assay (Miller et al., 2019). However, another study aligned with our observations, demonstrating that all culture-confirmed pathogens in bone and joint samples were confirmed by mNGS (Ruppe et al., 2017). There are several plausible explanations for the discrepancy in detection rate of mNGS assays conducted across specimen types. On the one hand, the relatively low pathogen detection rate reported for mNGS testing of CSF specimens in a previous study may reflect the fact that mNGS assay results in that study were compared to collective results obtained from standard culture, serological, and PCR testing (Miller et al., 2019), not only to results of culture testing alone. On the other hand, abscess specimens contain masses of killed bacteria and dead neutrophils (Miller and Cho, 2011). Despite the existence of a small number of pathogens that escape from phagosomes into the cell cytoplasm, the low viability of pathogenic organisms within abscesses negatively impacts pathogen recovery rates from abscess specimens. Thus, our data highlight the potential value of mNGS for detecting pathogens in abscess specimens, thereby ensuring early initiation of effective treatment and minimizing permanent loss of function or even death.

In addition to common microorganisms found in osteoarticular infections (Sigmund and McNally, 2019), we found that approximately half of our patient abscess specimens harbored 17 species of opportunistic microorganisms, a result that may reflect the high proportion of immunocompromised individuals in our study cohort. This experimental observation may provide valuable hints for improving clinical diagnosis of osteoarticular infections. First, a previous study demonstrated that culture isolation of low-virulence opportunistic microorganisms requires up to 14 days of culture vs. the 3 days needed for virulent microorganisms (Sigmund and McNally, 2019). Thus, collected abscess samples would likely need to be cultured for at least 2 weeks to recover clinically relevant opportunistic microorganisms. Second, the high frequency of detection of opportunistic microorganisms emphasizes that quality assurance should include strict collection and processing procedures to avoid sample contamination (Besser et al., 2018). Third, although targeted PCR has shown promising results for detection of pathogens in clinical specimens, commercial kits are focused primarily on detection of virulent pathogens rather than low-virulence organisms, further limiting clarity in pathogen detection. Conversely, unbiased detection of microbial sequences using mNGS allows identification of all viruses, bacteria, and fungi in a single standardized universal test for improved diagnosis of osteoarticular infections.

Although mNGS has the ability to greatly reduce turnaround time to within 2 days, the current diagnostic algorithm requires 7–10 days from specimen receipt for testing, due to involvement of third-party laboratories (Besser et al., 2018; Simner et al., 2018). For the most common microorganisms detected (MTBC), the PCR-based Xpert assay yielded comparable sensitivity and specificity to mNGS despite missed detection of organisms in two paucibacillary specimens; moreover, Xpert produced results within 2 h and detected MTB with RIF resistance, thereby improving timeliness of initiation of targeted therapy for osteoarticular TB patients (Zeka et al., 2011; Sun et al., 2019). As a consequence, here we recommend use of a laboratory diagnostic algorithm whereby conventional PCR-based assays would be used first to reveal the presence of common pathogens in osteoarticular infections; samples with negative results would then be tested *via* mNGS assay.

Interestingly, for two cases shown here to harbor MTBC and other microorganisms, MTBC sequences were not detected by mNGS assay. We speculate that a coinfecting microorganism within infected tissues of these patients may have inhibited growth and reproduction of MTBC, due to the latter’s slow growth rate (Verma et al., 1999). Consequently, only a small number of MTB reads was obtained that fell below the mNGS assay detection limit. Importantly, our data suggest that mixed infections may lead to adverse patient outcomes in spite of mNGS-guided treatment.

Our study had several obvious limitations. First, we retrospectively analyzed data of osteoarticular infections in inpatients of three TB-specialized hospitals. Thus, sampling bias stemming from retrospective design may have undermined our conclusions. Second, due to the potential for RNA degradation in storage and transportation steps, the mNGS assay used herein was not based on RNA sequencing, and thus excluded pathogens with RNA genomes from detection. Third, nucleic acid contamination can interfere with interpretation of results, especially among specimens with low microbial biomass (Simner et al., 2018). Despite the use of a no-template control throughout the entire mNGS workflow, we acknowledge the potential for false positive results due to contamination during specimen collection, processing, and testing. Fourth, human DNA contamination was not depleted during sample DNA purification, thus lowering the sensitivity of detection of non-human DNA. In addition, low numbers of non-human DNA sequence read hampered further analysis of mutations conferring drug-resistance. Fifth, although multiple commercial NGS platforms based on different technologies have been widely used to diagnose pathogens, here, only a single platform was used. Therefore, we could not compare accuracy rates of different NGS platforms for pathogen identification in specimens. Finally, the high out-of-pocket cost of mNGS should be considered before using this assay, and prospective studies should be carried out to assess the costs and benefits of mNGS use in clinical practice.

## Conclusion

In conclusion, our results demonstrate that mNGS has significantly greater pathogen detection rate in osteoarticular infections than conventional culture-based methods.

## Data Availability Statement

The datasets generated for this study can be found in the BIG GSA database, https://bigd.big.ac.cn/gsa-human/browse/HRA000256.

## Ethics Statement

The studies involving human participants were reviewed and approved by the Ethics Committee of Qingdao Chest Hospital. The patients/participants provided their written informed consent to participate in this study.

## Author Contributions

YP, MZ, and SQ designed the study. MZ, KT, FL, WZ, JF, GY, SQ, and YP participated in data collection. MZ, SQ, and YP participated in data analysis. MZ, KT, FL, SQ, and YP wrote the manuscript. Author ranking based on contributions to articles. All authors contributed to the article and approved the submitted version.

### Conflict of Interest

The authors declare that the research was conducted in the absence of any commercial or financial relationships that could be construed as a potential conflict of interest.

## Footnotes

^1^https://bigd.big.ac.cn/

^2^ftp://ftp.ncbi.nlm.nih.gov/genomes/
